# Sonographic Findings of the Bifid Median Nerve and Persistent Median Artery in Carpal Tunnel: A Preliminary Study in Chinese Individuals

**DOI:** 10.6061/clinics/2017(06)05

**Published:** 2017-06

**Authors:** Li Chen, Jie Chen, Bing Hu, Li-Xin Jiang

**Affiliations:** Department of Ultrasound in Medicine, Shanghai Jiaotong University Affiliated Sixth People’s Hospital, Shanghai Institute of Ultrasound in Medicine, Shanghai, China

**Keywords:** Sonography, Bifid Median Nerve, Persistent Median Artery, Anatomic Variation, Carpal Tunnel Syndrome

## Abstract

**OBJECTIVE:**

The aim of this study was to investigate the prevalence of anatomic variations of the bifid median nerve, persistent median artery and persistent median vein in Chinese individuals and their relationship with carpal tunnel syndrome.

**METHODS:**

One hundred and sixty median nerves were examined using ultrasonography and colour Doppler ultrasonography. The location, shape, and size of the bifid median nerve, persistent median artery and persistent median vein were recorded. The cross-sectional area of the bifid median nerve (two trunks) was measured at the level of the pisiform.

**RESULTS:**

Among the 160 wrists examined, a bifid median nerve was observed in 15 (9.4%) wrists, and a persistent median artery was observed in 12 (7.5%) wrists. These two variations either coexisted or were observed independently, and the probability of coexistence (6.3%) was higher than the probability of existing independently (bifid median nerve only 3.1%, persistent median artery only 1.3%). The cross-sectional area of the radial trunk was greater than (13 in 15, 86.7%) the cross-sectional area of the ulnaris trunk. Persistent median vein was observed in 9 wrists (5.6%).

**CONCLUSIONS:**

The persistent median artery and bifid median nerve tend to coexist, and the persistent median vein sometimes runs parallel to the persistent median artery. Their positional relationship in carpal tunnel is uncertain, and thus, preoperative ultrasound is necessary. These three variations do not present any additional risk for the development of carpal tunnel syndrome.

## INTRODUCTION

In 1977, Lanz 1 described the bifid median nerve as an anatomic variation in the median nerve. This condition occasionally coexists with a persistent median artery in carpal tunnel syndrome 2,3. A persistent median artery is occasionally accompanied by persistent median veins. Thus, it is important for surgeons to be aware of these structural variations when planning carpal tunnel release or other surgeries associated with carpal tunnel syndrome (CTS). Several studies have implicated bifid median nerve and persistent median artery as potential risks for CTS 4-7. However, this hypothesis has been disputed. Moreover, there are no studies concerning these anatomical variations in Chinese individuals.

Thus, in the present study, we assessed patients for the presence of bifid median nerve and persistent median artery in the forearm to the palm, analysed the prevalence of the anatomic variations of bifid median nerves and persistent median arteries in Chinese individuals and examined the position of the persistent median artery in relation to the median nerve.

## MATERIALS AND METHODS

### Study Participants

Participants were recruited regardless of gender and age, and all participants provided written, informed consent for the diagnostic procedures. The present study was approved by the ethics committee of Shanghai Jiao Tong University Affiliated Sixth People’s Hospital, and the investigations were performed according to Declaration of Helsinki on Ethical Principles.

The study included a total of 160 wrists from patients who underwent sonographic evaluations for any reason (including CTS) in the hospital from December 2014 to May 2015. CTS was clinically and electrophysiologically diagnosed if the patients had clinical symptoms of CTS.

Participants with diseases and conditions potentially associated with CTS, such as diabetes mellitus, hypothyroidism, rheumatoid arthritis, or amyloidosis, were excluded.

### Sonographic Evaluations

A fellowship-trained musculoskeletal radiologist performed the ultrasonographic and CDUS examinations using an Aplio500 Ultrasound System (Toshiba Medical Systems Co., Ltd., Tokyo, Japan) equipped with an 18-MHz high frequency linear array probe in real time.

The subjects were seated with the arm supported to maintain the position of the wrists and fingers extended, the forearm supinated, and the elbows and shoulder flexed. Each hand involved in the study was placed on a hard surface in a neutral position.

The course of the median nerve was consecutively evaluated from the forearm to palm in the axial plane. Nerves branching proximal to or within the carpal tunnel were considered bifid. Subsequently, transverse images of the median nerve were obtained at the level of the pisiform. The transducer was maintained perpendicular to the nerve to obtain better images. No additional force was applied to avoid any artificial nerve or vessel deformities.

Using CDUS, the persistent median artery was detected as a hypoechoic tubular structure with a pulsatile flow pattern accompanying the median nerve. Persistent median veins were defined as thin-walled tubular hypoechoic compressible structures running parallel to the persistent median artery. The presence of accessory vessels and the anatomic relationship of these structures to the median nerve were recorded. The diagnosis of CTS was based on the clinical history and physical and electrophysiological examinations of the patient.

### Statistical Analysis

Statistical analyses were performed using the Chi square test. The level of significance was set at *p*<0.05, and all analyses were performed using SPSS, version 17.0. Statistical analyses were performed to assess the prevalence of bifid median nerves and persistent median arteries in the study population to evaluate whether sex or location are correlated with this prevalence and to determine whether these two variations co-exist or exist independently.

## RESULTS

**Graph 1:**
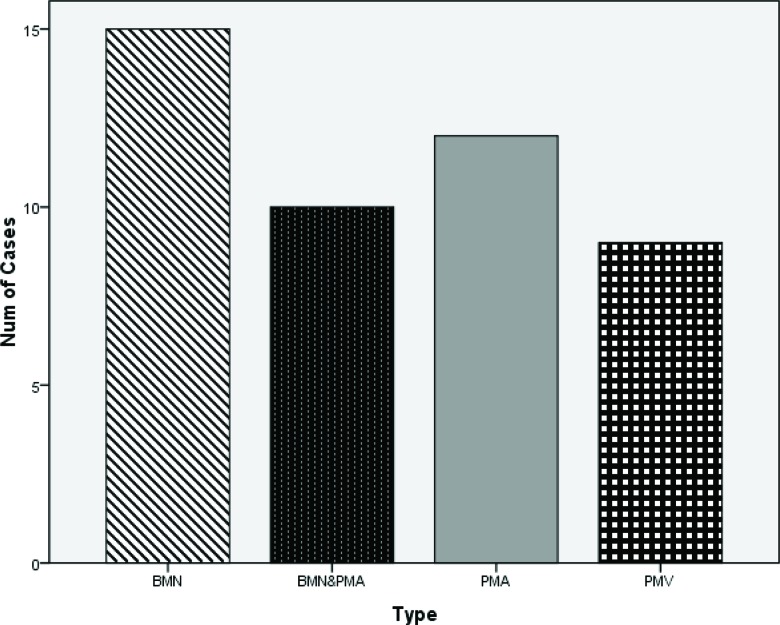
A bifid median nerve was observed in 15 wrists. A persistent median artery was observed in 12 wrists. Persistent median veins were observed in 9 wrists accompanied with PMA. PMA tended to accompany with BMN. PMV did not exist independently, which was always observed to be accompanied with PMA.

Among the 50 female patients and 30 male patients, a bifid median nerve was observed in 15 wrists (9.4%): 9 patients (5 right and 4 left) exhibited unilateral bifid median nerves, and 3 patients exhibited bilateral bifid median nerves. A persistent median artery was observed in 12 wrists (7.5%): 4 patients (4 right) exhibited unilateral vessel anomalies, and 4 patients exhibited bilateral vessel anomalies. Persistent median veins were observed in 9 wrists accompanied with PMA (5.6%) in Graph 1. There were no observable differences between the groups (female *vs*. male, left *vs*. right hand, and unilateral *vs*. bilateral), as presented in Table 1.

A single variation of the bifid median nerve was observed in 5 wrists. A single variation of the persistent median artery was observed in 2 wrists. A persistent median artery with a concurrent bifid median nerve was observed in 10 wrists. Significantly, in most cases, other than the persistent median artery or bifid median nerve, the persistent median artery typically runs in parallel with the bifid median nerve, which existed alone in carpal tunnel (Table 2).

**Figure 1 and Figure 2:**
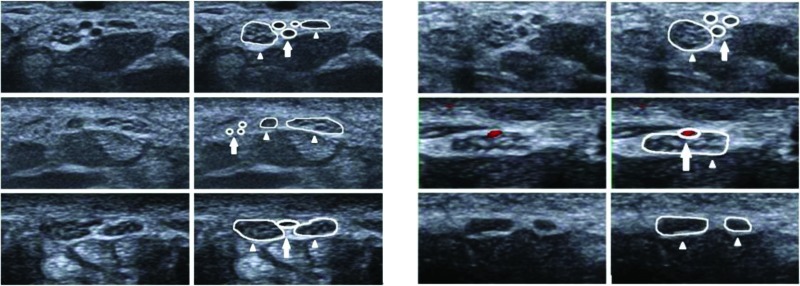
Transverse ultrasonographic image (left side) and corresponding outline-marked image (right side) of a bifid median nerve (thin arrow) and PMA (thick arrow) at the level of the proximal carpal tunnel. Different PMA positions in relation to the median anatomy and a bifid median nerve with an intermediate position in relation to the PMA were most frequently observed.

When in parallel with a bifid median nerve, the persistent median artery can be visualized in the middle of the two branches of the bifid median nerve or on the ulnar side of the ulnaris trunk. When running parallel to the median nerve, the persistent median artery was detected on the ulnar side or on the surface of the median nerve (Figure 1 and Figure 2).

**Graph 2:**
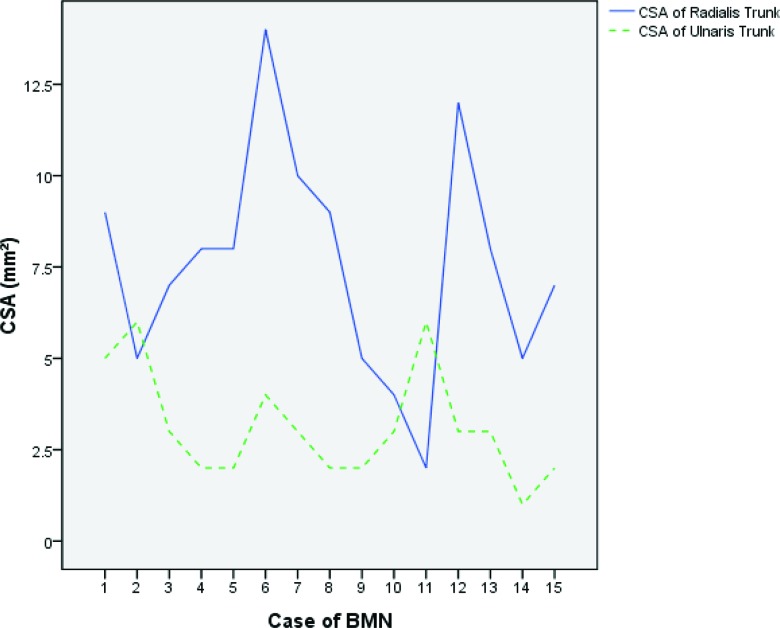
According to the measured results, the radialis trunk was larger than the ulnaris trunk in most cases (13 in 15).

Among the 15 cases of bifid median nerve, 12 cases were asymptomatic. Only 3 participants with bifid median nerve were both clinically and electrophysiologically diagnosed with CTS. A total of 9 cases of persistent median veins were accompanied with persistent median artery. We also observed that the radialis trunk was larger than the ulnaris trunk in most cases (13 in 15, 86.7%, Graph 2).

## DISCUSSION

The median nerve is the only nerve that runs through the carpal tunnel. In most individuals, the median nerve is observed as a singular nerve structure proximal to and within the tunnel and is divided into several branches that exit the tunnel 2. However, the bifid median nerve is an anatomic variation of the median nerve, which occasionally divides either proximal to or within the tunnel.

Both magnetic resonance imaging (MRI) and ultrasonography are used to detect median nerve anomalies, and the use of ultrasonography and MRI to detect carpal tunnel has been recently reported 8-10. Ultrasonography is recognized as a useful imaging method to visualize internal body structures, including tendons, muscles, nerves, joints, vessels and internal organs for potential pathology. The practice of dynamically examining the musculoskeletal system using ultrasound in real time is widely used. The use of sonography at high frequencies has been demonstrated to be a useful diagnostic tool in patients with CTS 10-12. The small structures of a bifid median nerve and the relationship of this structure to a persistent median artery can be observed more clearly using an 18-MHz high-frequency transducer.

Previous studies and several case reports have suggested that bifid median nerve occurs relatively frequently in patients with CTS 4-7. However, this conclusion is controversial. Indeed, Granata et al. suggested that bifid median nerves frequently occur in both CTS patients and in the general population. Their study showed that it has a similar prevalence in the CTS (18.5%) and control groups (15.4%) 13. In the present study, a bifid median nerve was detected in 9.4% of wrists (160 wrists). This rate is consistent with the detection in 8.6% of wrists as observed by Walker et al. 14.

Most participants were asymptomatic, and no electrophysiological findings were observed. Only 3 patients with a bifid median nerve were also diagnosed both clinically and electrophysiologically with CTS. The pathogenic causes were determined to be Colles fractures, ganglion in the CT, and idiopathic. There is no evidence that a bifid median nerve results in CTS. Thus, a bifid median nerve cannot be considered as a risk factor for developing CTS.

Moreover, in most cases (86.7%, 13 in 15), we observed that the radialis trunk was larger than the ulnaris trunk. It has recently been reported that the radialis trunk of the bifid median nerve results in CTS 2, but the study did not depict the CSA of the radialis or ulnaris trunk. Thus, we propose that the radialis trunk was larger than the ulnaris trunk in that case, making it easier to associate this variation with CTS rather than the ulnaris trunk.

**Figure 3:**
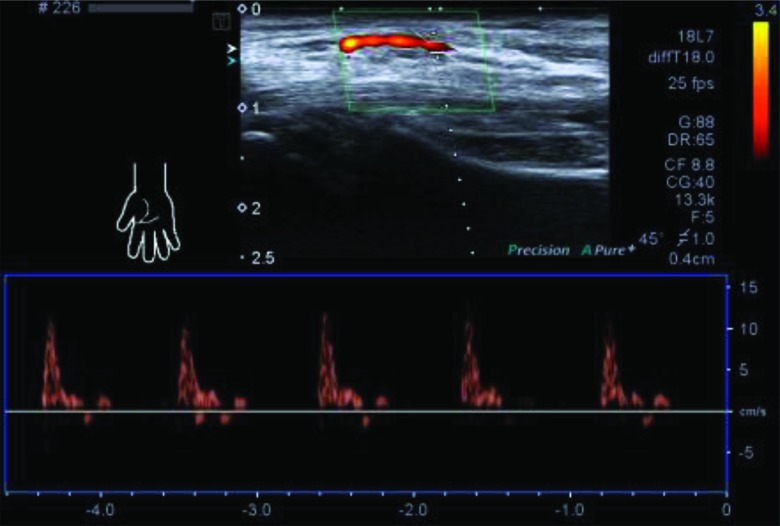
Spectral Doppler waveform of a PMA proximal to the carpal tunnel, which demonstrate peripheral arterial flow. The artery can be visualized in the middle of the two branches of a bifid median nerve.

We also assessed the prevalence of a persistent median artery within the carpal tunnel in the same population. The median artery develops from the axillary artery, runs parallel to the median nerve, and typically regresses in the second embryonic month. In some individuals, the median artery does not undergo reduction but persists throughout the entire lifespan as a sizable vessel, called the persistent median artery 2. In the present study, a persistent median artery was detected in 6.9% of the population (160 wrists) using ultrasonography and CDUS. A CDUS examination also revealed an arterial blood flow wave (Figure 3).

Persistent median arteries 1.0 to 1.5 mm in diameter are typically asymptomatic. There is no evidence demonstrating a persistent median artery resulting in CTS. Importantly, a persistent median artery could be an independent risk factor for CTS when enlarged to 3 mm in diameter in some pathological conditions, including internal thrombus, aneurysm, and calcified plaque formation 15-17. Thus, the early detection of the thrombosed median artery using ultrasonography can result in prompt anticoagulant therapy, thus preventing the need for any surgery or thrombolytic therapy 10. Although Pierre-Jerome, et al. observed that the PMA was more frequently observed on the left side 9, no significant differences between the left and right side of the wrists were observed in the present study. We also detected the coexistence of a persistent median artery with a bifid median nerve in the carpal tunnel in most cases. This situation occurred more frequently than the independent existence of a persistent median artery. The position of the bifid median nerve and the persistent median artery in the carpal tunnel varied in the present study. When running parallel with the bifid median nerve, the persistent median artery can be visualized in the middle of the two branches of the bifid median nerve or on the ulnar side of the ulnar trunk. When running parallel with the median nerve, the persistent median artery can be detected on the ulnar side or on the surface of the median nerve. Thus, patients should undergo ultrasound scanning of the wrists prior to wrist surgery to avoid accidentally injuring these anatomic variations.

In many cases, a bifid median nerve was accompanied by persistent median veins in addition to persistent median artery. Compared with previous studies, a higher prevalence of persistent median veins accompanied by a persistent median artery and bifid median nerves has been detected in wrists with carpal tunnel syndrome, reflecting the use of an 18-MHz high-frequency probe and more appropriate methods. Persistent median veins were defined as thin-walled and compressible structures.

**Figure 4:**
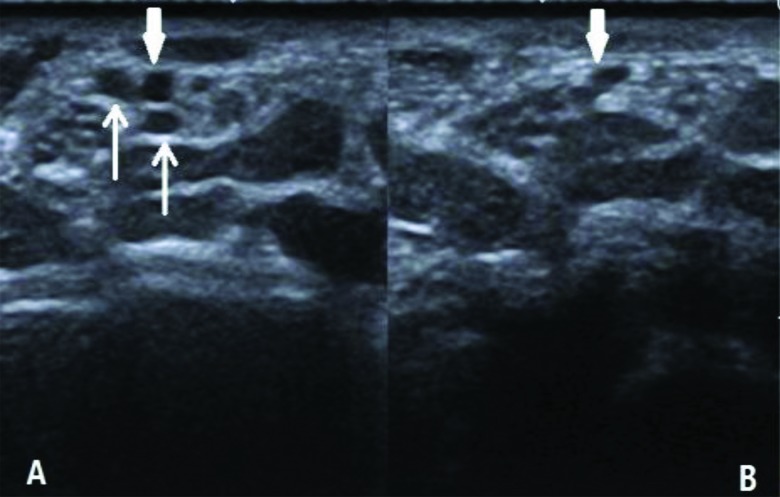
Persistent median veins are often accompanied with PMA (in Figure 4A, thin arrow). The veins become deformed and invisible (Figure 4B) when additional force is applied to the surface of the wrist during the ultrasonography examination. A persistent median artery is difficult to deform (thick arrow).

When additional force is applied to the surface of the wrist during the ultrasonography examination, the vessels become deformed and invisible (Figure 4). To improve the detection of a persistent median vein, each hand involved in the study was placed on a hard surface in a neutral position, with adequate coupling agent and the holding probe placed gently on the wrist. We deduced that some persistent median veins may not be detected in previous studies, although these structures were likely present. We also assumed that an internal thrombus in a persistent median vein could be a new risk factor for CTS, similar to the persistent median artery. It is important for surgeons to be aware of the existence of these conditions (BMN, PMA, and PMV) prior to carpal tunnel release. In specific pathological conditions, these anatomical variations may be the primary causes for the development of CTS. Thus, it is necessary for patients to undergo ultrasonographic examination of the wrist prior to surgery to avoid the risk of nerve and blood vessel injury.

Bifid median nerve and persistent median artery do not present additional risks for developing CTS. Thus, more attention should be given to persistent median veins and persistent median artery, which may represent new pathogenic factors for CTS when internal thrombi form in some pathological conditions. Ultrasonographic examination prior to wrist surgery could prevent accidental injury due to these variations. This is a preliminary research study in Chinese individuals, and thus, it is necessary to amplify samples in future studies.

## AUTHOR CONTRIBUTIONS

Chen L performed the study, was responsible for the data analysis and manuscript drafting. Chen J designed the study and helped to draft the manuscript. Hu B and Jiang LX helped to revise the manuscript. All of the authors reviewed the manuscript.

## Figures and Tables

**Table 1:** No Differences between the Groups were Observed (Female *vs*. Male, Left *vs*. Right Hand, and Unilateral *vs*. Bilateral).

	BMN either	BMN neither	*p-*value	PMA either	PMA neither	*p-*value
**Gender**			0.824			0.120
**Male**	6 (20.0)	24 (80.0)		7 (23.3)	23 (76.7)	
**Female**	9 (18.0)	41 (82.0)		5 (10.0)	45 (90.0)	
**Location**			0.786			0.369
**Left Hand**	7 (8.8)	73 (91.2)		4 (5.0)	76 (95.0)	
**Right Hand**	8 (10.0)	72 (90.0)	0.131	8 (10.0)	72 (90.0)	1.000
**Unilateral**	9 (11.3)	71 (88.7)		4 (3.8)	76 (96.2)	
**Bilateral**	3 (3.8)	77 (96.2)		4 (5.0)	76 (95.0)	

**BMN**: bifid median nerve; **PMA**: persistent median artery

**Table 2:** The Probability of the Coexistence of these Two Variations was Higher than the Probability of the Variations Existing Independently.

	Yes	No	*p*-value	OR	95% CI
**BMN only**	5 (3.1%)	155 (96.9%)	0.290	0.484	(0.162-1.449)
**PMA with BMN**	10 (6.3%)	150 (93.7%)			
**PMA only**	2 (1.3%)	158 (98.7%)	0.035	0.190	(0.041-0.881)
**PMA with BMN**	10 (6.3%)	150 (93.7%)			

**BMN:** bifid median nerve; **PMA:** persistent median artery
